# Pediatric Crohn's disease diagnosis aid *via* genomic analysis and machine learning

**DOI:** 10.3389/fped.2023.991247

**Published:** 2023-03-23

**Authors:** Zhiwei Zheng, Sha Zhan, Yongmao Zhou, Ganghua Huang, Pan Chen, Baofei Li

**Affiliations:** ^1^ Department of Pediatrics, Zhongshan Hospital of Traditional Chinese Medicine Affiliated to Guangzhou University of Traditional Chinese Medicine, Zhongshan, China; ^2^ School of Chinese Medicine, Jinan University, Guangzhou, China; ^3^ Department of Pediatrics, The First Affiliated Hospital of Guangzhou University of Traditional Chinese Medicine, Guangzhou, China

**Keywords:** pediatric Crohn’s disease, machine learning, diagnostic model, artificial neural network, immune cell cells

## Abstract

**Introduction:**

Determination of pediatric Crohn's disease (CD) remains a major diagnostic challenge. However, the rapidly emerging field of artificial intelligence has demonstrated promise in developing diagnostic models for intractable diseases.

**Methods:**

We propose an artificial neural network model of 8 gene markers identified by 4 classification algorithms based on Gene Expression Omnibus database for diagnostic of pediatric CD.

**Results:**

The model achieved over 85% accuracy and area under ROC curve value in both training set and testing set for diagnosing pediatric CD. Additionally, immune infiltration analysis was performed to address why these markers can be integrated to develop a diagnostic model.

**Conclusion:**

This study supports further clinical facilitation of precise disease diagnosis by integrating genomics and machine learning algorithms in open-access database.

## Introduction

Increasing incidence rates of pediatric Crohn's disease (CD) over the past decades have been reported in relevant studies (1, 2). Pediatric CD present heterogeneous threat to the health of children, with growth retardation, metabolic bone disorders, bone density reduction and pubertal delay (3–5). Beyond clinical manifestation, endoscopic and histological examination are considered the most reliable technique for diagnosing CD. However, large inter- and intra-observer variability may exist in the subjective interpretation of endoscopic and histopathologic appearance. Mislabeling occurs frequently, and a fraction of pediatric CD is deemed ulcerative colitis incorrectly (6). The onset of pediatric CD is insidious, and strictures or penetrating disease have already occurred at diagnosis in some children (7). Additionally, up to 50% of pediatric CD require intestinal resection within 10 years after diagnosis (8). Hence, accurate diagnosis during the initial stage of pediatric CD is crucial but challenging for rapid intervention and better prognosis.

Recent advancements made in machine learning and extensive use of RNA sequencing have enabled the construction of automated diagnostic model for knotty diseases. It has been confirmed that deep learning algorithms assisted doppler improved the classification of ovarian tumors (9). Ultrasound and machine learning approaches have been used for the differential diagnosis on melanocytic lesions patients (10). Moreover, using random forest (RF), weighted gene correlation network analysis (WGCNA), least absolute shrinkage and selector operation (LASSO) and support vector machine-recursive feature elimination (SVM-RFE), gene expression profile can be used to identify biomarkers associated with classification tasks such as cancer detection, recurrence prediction, prognosis prediction, and severe sepsis detection (11–13). Though enormous novel biomarkers heretofore have not been recognized as disease-associated, they may be extracted to build diagnostic model by deep-learning supervision based on diagnostic label in recent work (14, 15).

Though like adults, currently the diagnosis of pediatric CD is mainly based on clinical manifestations and digestive endoscopy, in the broader context, childhood- onset CD may have more complex pathogenesis that is driven by gene defects (16). With the advent of next-generation sequencing and application of molecular biomarkers, diagnosis for many genetically related diseases have become increasingly accurate and timely (17, 18). For instance, mutations in TNFRSF13B, NFKB1, NFKB2, CTLA4 and STAT3 are indications for the early molecular diagnosis of patients with predominantly antibody deficiency such as predominantly antibody deficiencies (19). Genetic aberrations such as PMP22, GJB1, MFN2, MPZ, SH3TC2 and GDAP1 mutations identified by targeted next-generation sequencing panels are able to perform effective diagnosis in previously undiagnosed and rare subtypes of Charcot-Marie-Tooth disease (20). Using machine learning, large amounts of biomedical data such as genome, transcriptome and proteome have been investigated to identify underlying causative factors and relative biomarkers behind complex illnesses (21). Recent studies performed on high-throughput data from GEO and TCGA datasets have developed various diagnostic models *via* investigating candidate genes, which can assist in discovering biomarkers and diagnosing for different kinds of diseases (22, 23).

## Materials and methods

### Collection of GEO datasets

Publicly available data from the Gene Expression Omnibus (GEO) databases (https://www.ncbi.nlm.nih.gov/geo/) was collected with the following key terms: “Crohn's disease”, “inflammatory bowel diseases (IBD)” and “child or children or pediatric”. Result of data retrieval was filtered by “expression profiling by high throughput sequencing” in “Homo sapiens”. All relevant reference lists were reviewed manually for further identification. Only datasets that met the following conditions were included: (1) All cases were pathologically diagnosed as Crohn's disease and the controls were normal intestinal tissues. (2) The minimum sample size of cases and controls was 10. And Exclusion criteria were: ulcerative colitis, miRNA analyses, duplicate. Eventually, the raw and series matrix data of available datasets were downloaded and summarized in Table 1.

**Table 1:** Gene expression datasets from GEO database.

Database	Samples	Platforms	Contributor
GSE57945	218 pediatric CD vs. 42 non-IBD	GPL11154 Illumina HiSeq 2000	Yael Haberman (24, 25)
GSE93624	210 pediatric CD vs. 35 non-IBD	GPL11154 Illumina HiSeq 2000	Urko M Marigorta (26)
GSE101794	254 pediatric CD vs. 50 non-IBD	GPL6365 DKFZ Homo sapiens 8k BAC-array version 2	D E Stange (27)
GSE117875	6 pediatric CD vs. 7 non-IBD	GPL16791 Illumina HiSeq 2500	Daniel Kelly (28)
GSE62207	259 pediatric CD vs. 51 non-IBD	GPL11154 Illumina HiSeq 2000	Yael Haberman (29)

### Study design and data processing

**Figure 1:**
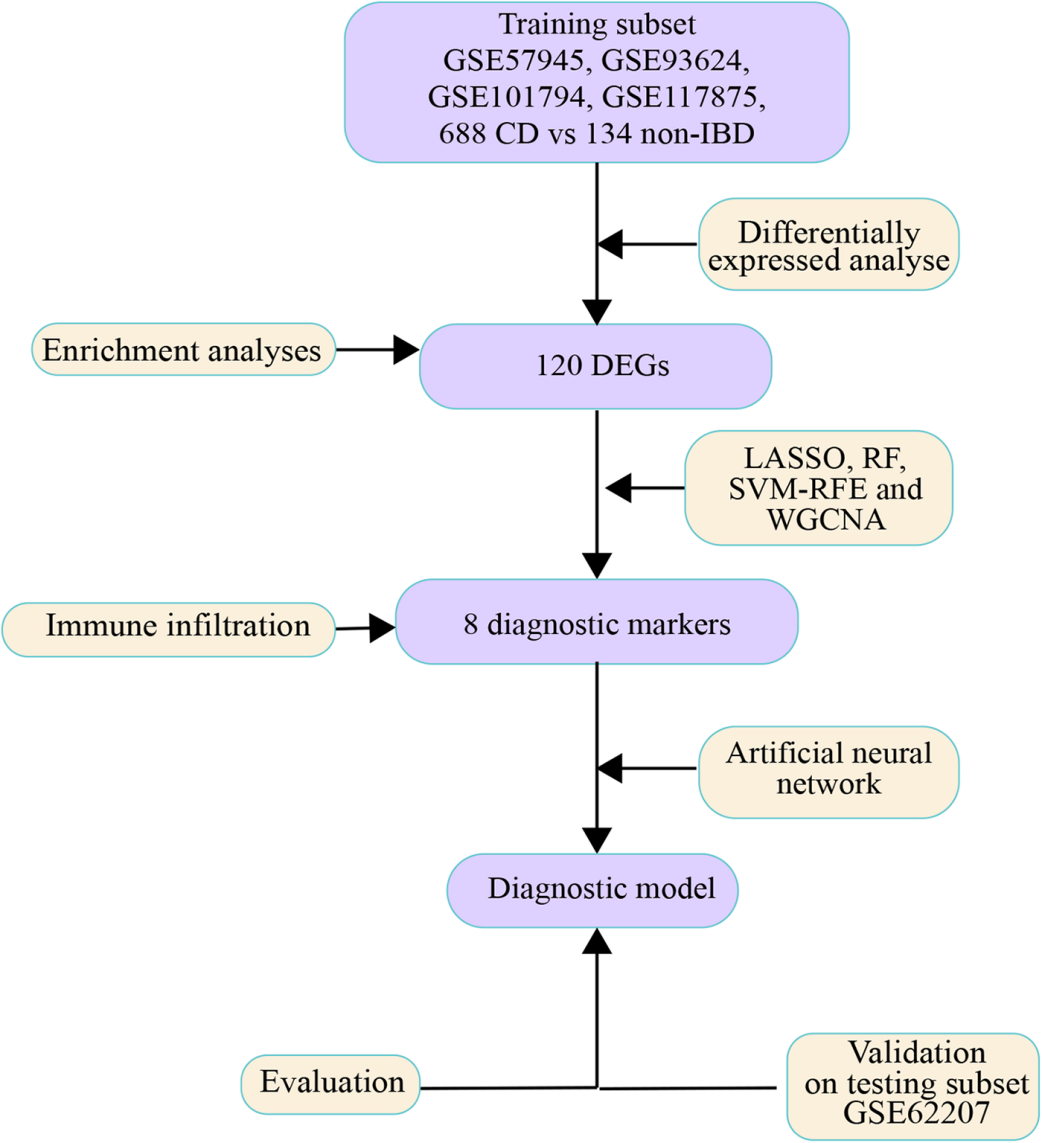
Workflow diagram. The flow diagram of whole process of data analysis.

The flow diagram is shown in Figure 1. Each probe expression matrix was extracted and then converted into a gene expression matrix from corresponding series matrix data using Perl 5.36 (https://www.perl.org/). Raw data of datasets were extracted with affy package in R 4.1.1(https://www.r-project.org/). Extracted expression data were normalized and converted to log2-based logarithms using the rma tool of affy package in R 4.1.1. Datasets GSE57945, GSE93624, GSE101794 and GSE117875 were merged into a metadata cohort and served as training subset. ComBat function of sva package in R 4.1.1 was run to remove batch effects from expression matrixes (30). In addition, dataset GSE62207 was served as testing subset.

### Identification of differentially expressed genes

Differentially expressed genes (DEGs) in pediatric CD vs. non-IBD were identified with empirical Bayesian method of limma R-package. Benjamini and Hochberg false discovery rate (FDR) and cut-off of log2 fold change (log2FC) were applied to balance both discovery of statistically significant genes and limitations of false-positives. The threshold for DEGs were set to log2FC > 1 and FDR <0.05. All DEGs were uploaded to the STRING database (https://www.string-db.org/). The minimum required interaction score was set to 0.900. The interactive relationship between DEGs was screened from the protein level, and the protein–protein interaction (PPI) data of DEGs were downloaded for construction of a PPI network in Gephi software (version 0.9.6).

### Functional enrichment analyses and annotation

Gene names of DEGs were converted to gene ID by org.Hs.eg.db R-package. Enrichment analyses were carried out in clusterProfiler R-package to explore the functions and pathways enriched by DEGs (31). Gene Ontology (GO) biological processes and Kyoto Encyclopedia of Genes and Genomes (KEGG) pathway analysis were considered. Biological processes and signal pathways with a *p*-value <0.05 were considered significant. In order to clarify the gene expression level of significantly enriched functional pathways more intuitively, Gene set enrichment analysis (GSEA) was performed with GSEA tool of clusterProfiler R-package. Disease Ontology (DO) analysis was performed to annotate potential similarities among DEGs in disease context using DOSE R-package. The above results were visualized by enrichplot and ggplot2 R-package. Metascape analysis was performed on the Metascape web platform (https://metascape.org) to further verify the function enrichment of DEGs, and *p*-value <0.05 was set as the cutoff value.

### Identification of diagnostic markers

LASSO, RF and SVM-RFE algorithms were used to filter diagnostic markers base on training subset with R-package glmnet, randomForest and e1071, respectively. The optimal diagnostic markers were selected by the LASSO algorithm with ten-fold cross validation, and the weight of the LASSO penalty was represented by *λ*. Herein, the *λ* = 0.0009077964 was selected as optimal value *via* minimum criteria (32). RF classification initialized with 500 trees was used to classify the diagnostic result of each sample (33, 34). The importance of markers was calculated by the Gini impurity values. The top-ranked 100 genes were then selected as the diagnostic markers. SVM-RFE algorithms was conducted based on radial basis function and 10-fold cross-validation (CV). According to the minimum CV error (minimum CV error = 0.2270997), 28 diagnostic markers were selected (35). WGCNA is a method to screen co-expressed gene modules. A co-expression network of DEGs was constructed to extract diagnostic markers in disease-related modules using WGCNA R-package (36, 37). A scale-free network was built by a β-power operation. we chose the soft power *β* = 6. The similar gene expression was divided into several gene co-expression modules. There are at least 100 genes in each module. Subsequently, the module-trait correlation between modules and diagnosis was calculated. Then we chose the method of dynamic tree cutting to recognize co-expression gene modules. The module eigengene (ME) was calculated to quantify overall expression level of each module, and the Z-summary was calculated to estimate the conserved modules. Finally, the genes contained in module with high correlation coefficient were defined as the candidate markers. The intersection markers among LASSO, RF, SVM-RFE and WGCNA algorithms were defined as final diagnostic markers and exhibited in a Venn diagram generated by venn R-package. The clusters separability of diagnostic markers was observed in a heatmap drawn by pheatmap R-package.

### Development of artificial neural network diagnostic model

The training subset and testing subset were filtered and normalized by min-max normalization. An Artificial Neural Network (ANN) model of diagnostic markers was constructed by neuralnet R-package. Five hidden layers were set as the model parameter. The disease classification score is defined as the sum of the product of weight score multiplied by expression levels of the diagnostic markers. The architecture and connection between layers that mediate variable importance of the ANN model was visualized by the NeuralNetTools R-package.

### Evaluation of diagnostic efficacy

Both training subset and testing subset were used to measure the ability of each diagnostic marker to classify the pediatric CD samples. A five-fold cross-validation of the ANN model was performed by the confusion matrix function of Caret R-package in training subset. And classification of ANN model for pediatric CD samples was tested on the testing subset for further verification of effectiveness. All classification performance were drawn into ROC curves by pROC R-package and the areas under the curves (AUC) were compared.

### Immune analysis algorithm and correlation between immune cells and diagnostic markers

CIBERSORT is one of deconvolution algorithms that combine the labeled genomes of different immune cell subpopulations to calculate the proportion of LM22 leukocyte in tissues. LM22 gene signature matrix was downloaded from CIBERSORT website (https://cibersortx.stanford.edu/download.php). All datasets were merged into a metadata cohort and batch effects of all datasets were removed. CIBERSORT R script v1.04 (https://rdrr.io/github/zy26/SSMD/src/R/CIBERSORT modified.R) was run to calculate the score of each immune cell base on the merged dataset. Non-parametric correlations were used to determine the correlation between diagnostic markers and immune cells.

## Results

### Screening of DEGs in training datasets

**Figure 2:**
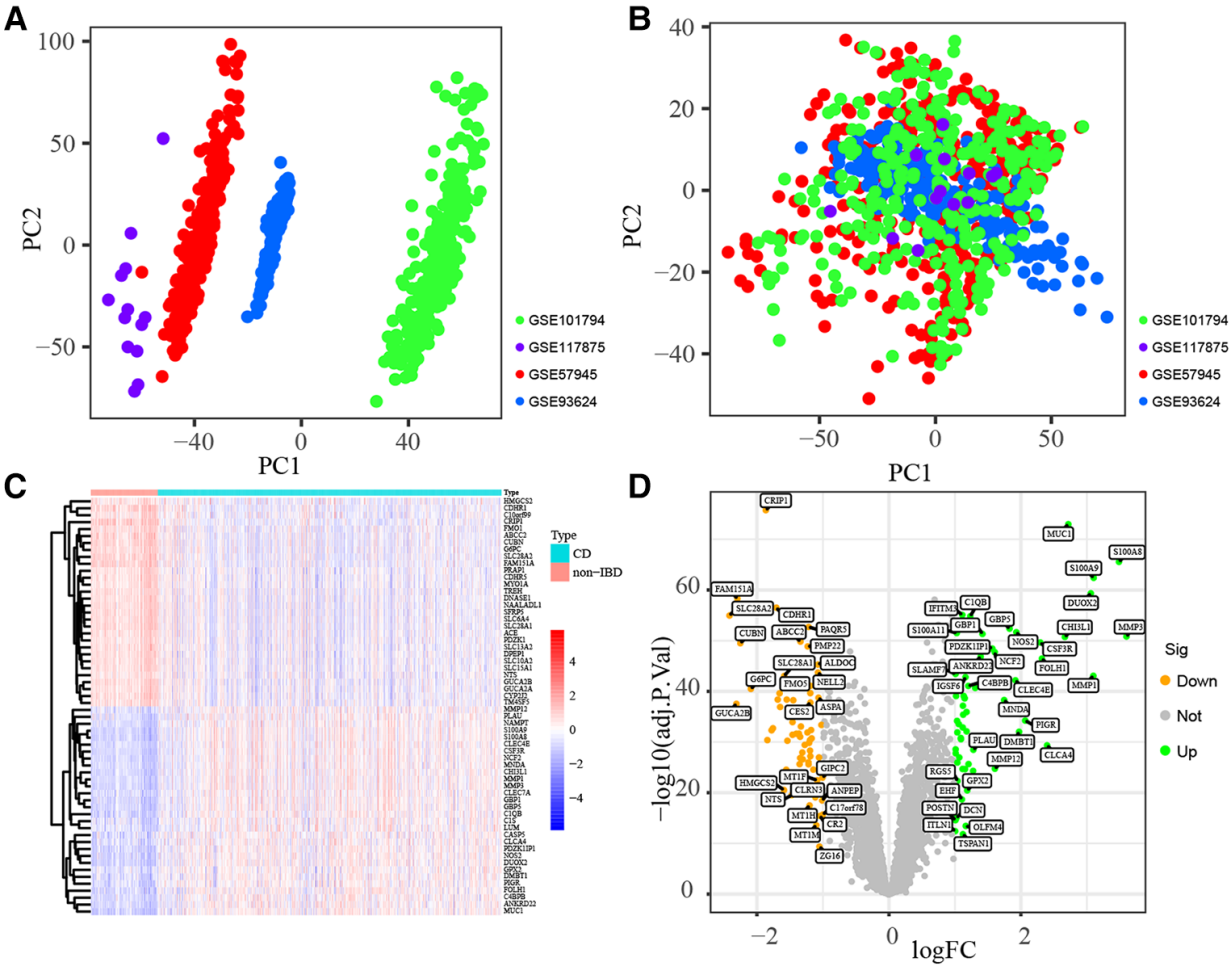
Identification of DEGs. Principal component analyses (PCA) performed to remove batch effect and in training subset. Before batch correction (**A**) and after batch correction (**B**). Identification of DEGs in training subset. Hellman (**C**) and Volcano plot (**D**).

Datasets GSE57945, GSE93624, GSE101794 and GSE117875, including 688 pediatric CD and 134 non-IBD samples, were merged into training subset. The training subset before (A) and after (B) batch correction was presented in Figure 2, which indicated that the batch effect in the training dataset was removed successfully. As shown in Figures 2C,D, 120 DEGs including 59 up-regulated and 61 down-regulated genes were identified (All up-regulated and down-regulated genes were listed in Supplementary Table S1).

### Functional enrichment analysis and classification of hub proteins

**Figure 3:**
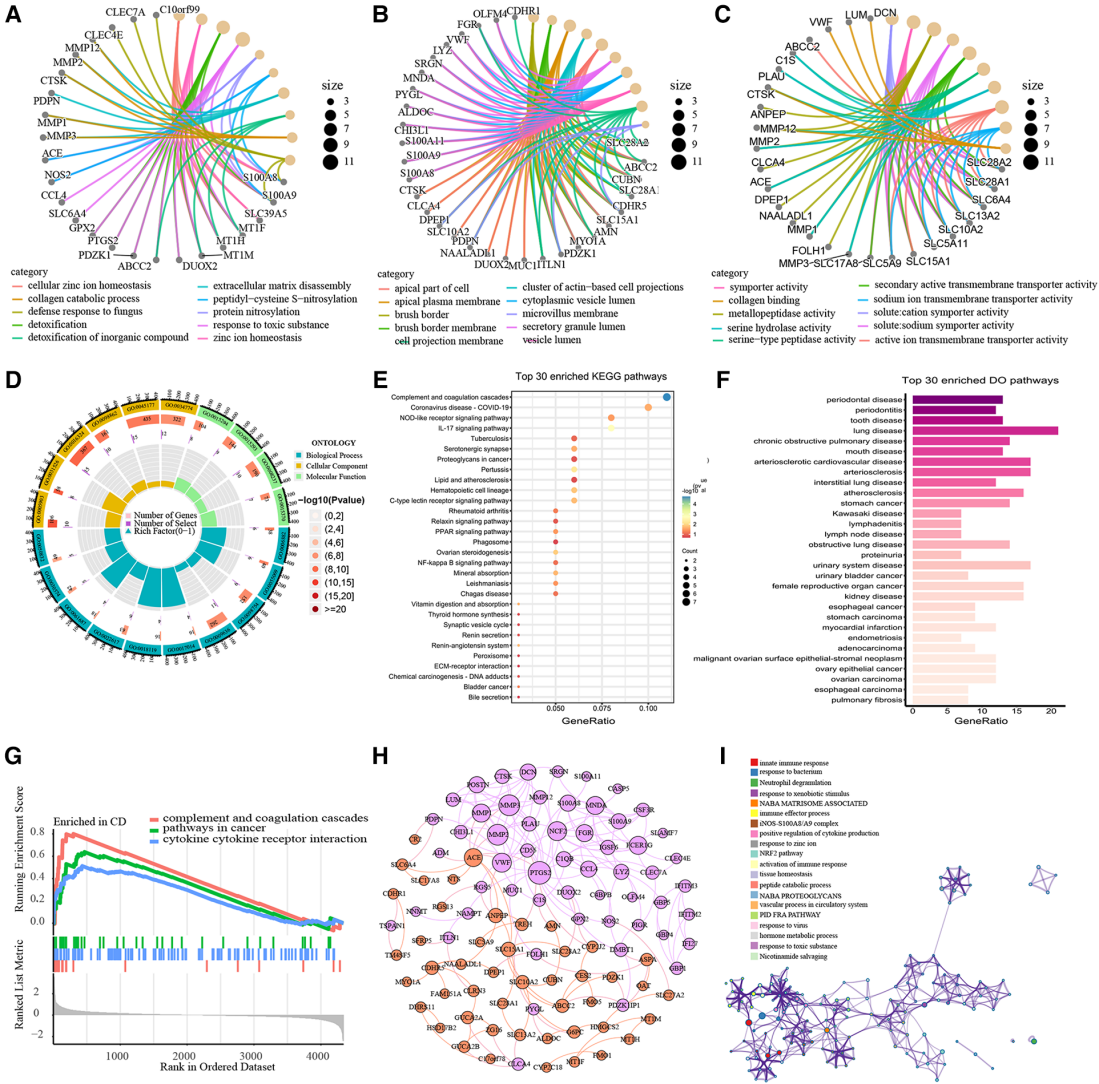
Functional Enrichment Analysis of DEGs. The top 10 ranked GO enrichment terms of BP (**A**), CC (**B**) and MF (**C**). Chord diagram of the top 20 ranked GO enrichment terms (**D**). Bubble chart of KEGG pathway analysis (**E**). Bar plot of Disease Ontology analysis (**F**). GSEA plot of the pathways enriched in pediatric-CD (**G**). The PPI network of DEGs. The purple nodes represent up-regulated DEGs and the orange nodes represent down-regulated DEGs. The size of nodes indicates the degree value of the DEGs. (**H**). Network of enriching terms in Metascape analysis of DEGs, colored by cluster ID. The nodes that share the same cluster ID are typically close to each other (**I**).

GO and KEGG functional enrichment analyses were performed to investigate the biological features of the 120 DEGs. GO functional enrichment result revealed 220 terms of up-regulated DEGs and 105 terms of down-regulated DEGs respectively across BP, CC and MF categories (Supplementary Table S2). All DEGs were enriched in the cellular zinc ion homeostasis, zinc ion homeostasis and response to the toxic substance in BP category, markedly (Figure 3A). Enriched CC terms included brush border, brush border membrane and apical plasma membrane (Figure 3B). In the MF category, genes were mainly enriched in solute cation symporter activity, symporter activity and metallopeptidase activity (Figure 3C). The top 20 ranked GO enrichment terms were displayed in Figure 3D. Moreover, pathways terms of KEGG pathway analysis are depicted in Figure 3E. The DEGs were chiefly enriched in Complement and coagulation cascades (hsa04610), IL-17 signaling pathway (hsa04657), Pertussis (hsa05133), Ovarian steroidogenesis (hsa04913) and Hematopoietic cell lineage (hsa04640). Top-ranked DO terms were listed in Figure 3F. Periodontal disease, periodontitis, tooth disease, lung disease and chronic obstructive pulmonary disease were all strongly enriched with respect to the DEGs. Finally, we performed a GSEA analysis on the 120 DEGs (Figures 3G). 5 pathways were enriched in pediatric CD, including complement and coagulation cascades, pathways in cancer and cytokine receptor interaction. To explore interactions and association pathways of DEGs, a PPI network was we constructed. PTGS2, MMP2, MMP3, VWF, NCF2, ACE, MMP1, FCER1G and MNDA were identified as the top 10 hub genes by the degree value (Figure 3H). Metascape analysis suggested that DEGs were mainly enriched in terms of innate immune response, response to bacterium, neutrophil degranulation, response to xenobiotic stimulus, Naba matrisome associated and immune effector process (Figure 3I).

### Identification of diagnostic markers

**Figure 4:**
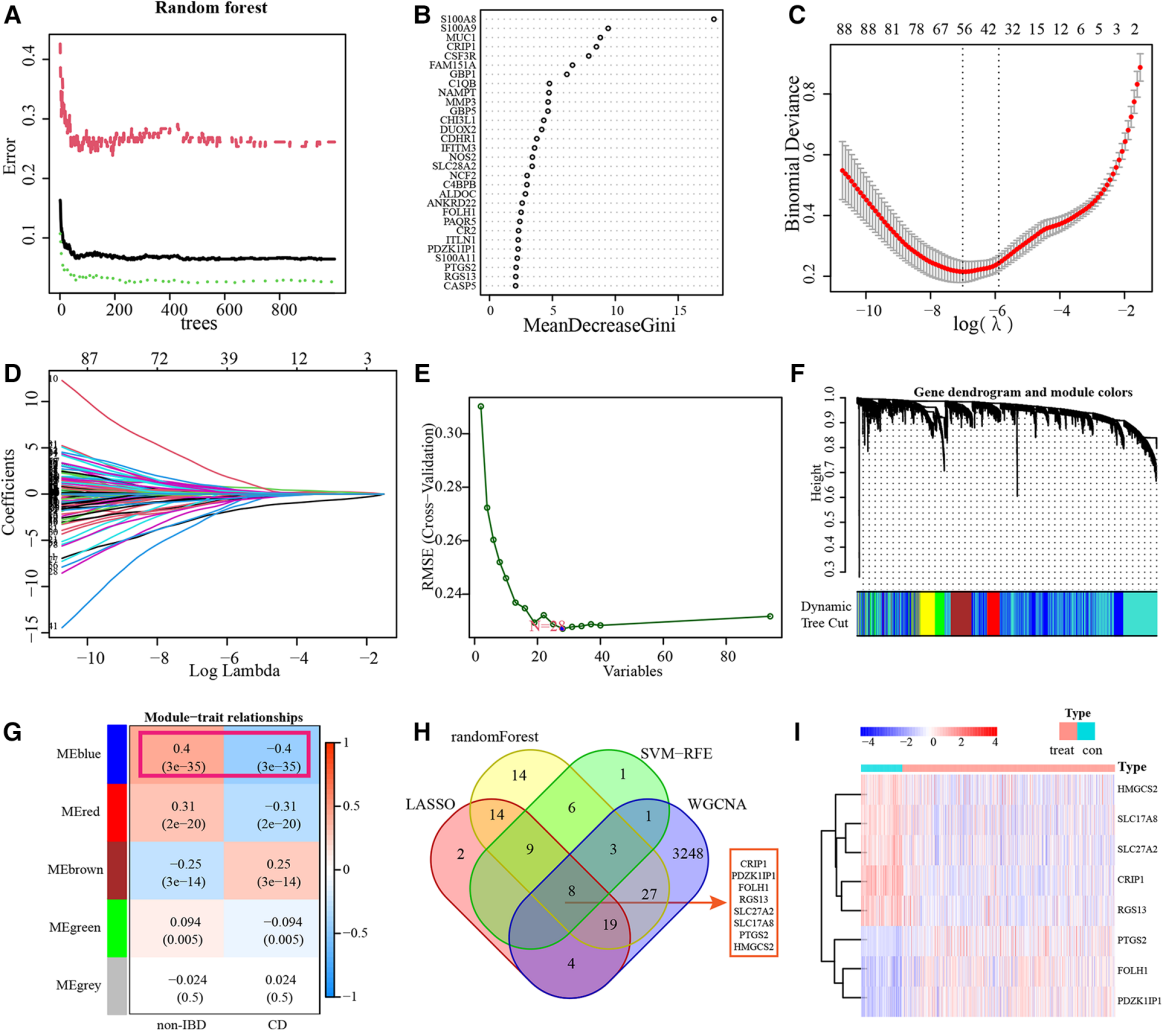
Identification of diagnostic markers. RF results of the relative importance of prognostic targets (**A,B**). LASSO regression analysis identified 56 markers to construct a diagnostic model (**C,D**). SVM-RFE algorithm screened 28 diagnostic markers in training datasets (**E**). WGCNA identified cluster dendrogram and co-expression modules. Each color represents one module (**F**). Correlation analysis between the gene module and the diagnosis of CD (**G**). 8 intersections of diagnostic markers of RF, LASSO, SVM-RFE and WGCNA (**H**). Heatmap of identification of 8 diagnostic markers in training subset (**I**).

RF Algorithm uncovered 100 prognostic targets of pediatric CD. The top-ranked 30 genes were displayed. And S100A8 was identified as the most important marker in terms of diagnostic (Figures 4A,B and Supplementary Table S3). The DEGs were applied to a LASSO regression analysis, and finally a machine learning model for the diagnosis of pediatric CD consisting of 56 gene markers was constructed (Figures 4C,D and Supplementary Table S3). A total of 28 genes were identified as diagnostic biomarkers based on SVM-RFE algorithm (Figure 4E and Supplementary Table S3). WGCNA was used to construct a co-expression network based on the average linkage method, and 5 modules were generated (Figures 4F,G). Among them, blue modules contained a total of 3,310 genes and showed the strong correlation with the diagnosis of pediatric CD (Supplementary Table S3). There are 8 overlapping genes including CRIP1, PDZK1IP1, FOLH1, RGS13, SLC27A2, SLC17A8, PTGS2 and HMGCS2 among RF, LASSO, SVM-RFE and WGCNA. The results of hierarchical clustering produced by 8 diagnostic markers were shown in the heatmap of unsupervised clustering (Figure 4I).

### Construction of the diagnostic model of artificial neural network

**Figure 5:**
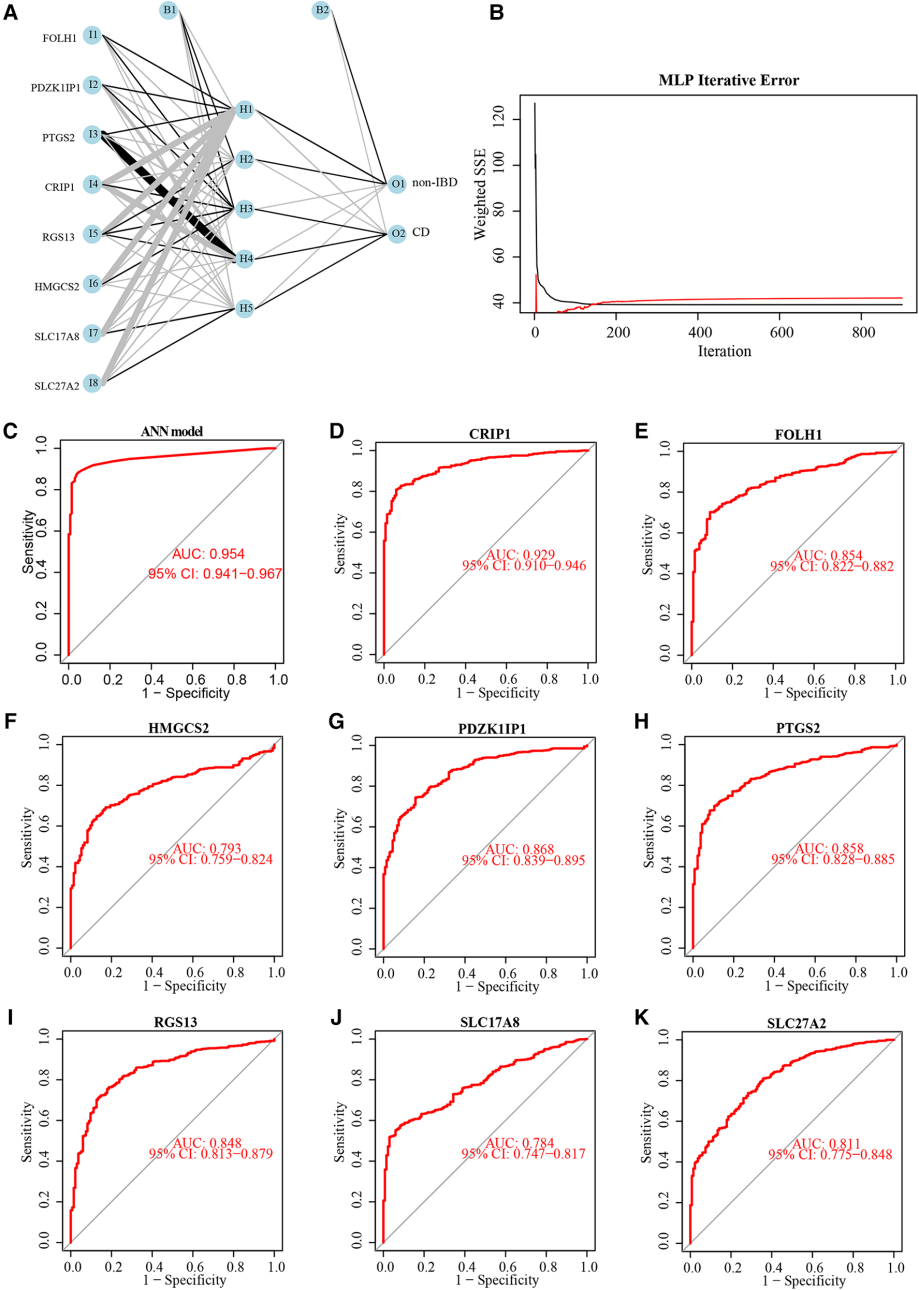
Neural network topology of the training subset, and performance evaluation of the diagnostic model and 8 diagnostic markers. Neural network topology of training subset with included an input layer with 8 neurons, a hidden layer with 5 neurons, and an output layer with 2 neurons (**A**). The cumulative error curves of the ANN model (**B**). Performance evaluation of the ANN model (**C**) and 8 diagnostic markers (D-K) by ROC curves and their AUC values.

The weight of 8 diagnostic markers was calculated by ANN analysis based on training subset to construct a diagnostic model furtherly. The ANN model for classifying the markers expression data between non-IBD and CD included an input layer with 8 neurons, a hidden layer with 5 neurons, and an output layer with 2 neurons (Figure 5A). It is obvious that the prediction error of ANN model decline to a stable level quickly, with the increase in the training iterations (Figure 5B). The weight of each diagnostic marker in ANN model was detailed in Supplementary Table S4. A ROC curve was generated to show the validation results based on training subset, which displays the model classification performance initially (Figure 5C). The ROC curves were used to show the classification efficiency of 8 diagnostic markers, and each AUC was compared (Figures 5D–K).

### Validation of ANN model based on testing subset

**Figure 6:**
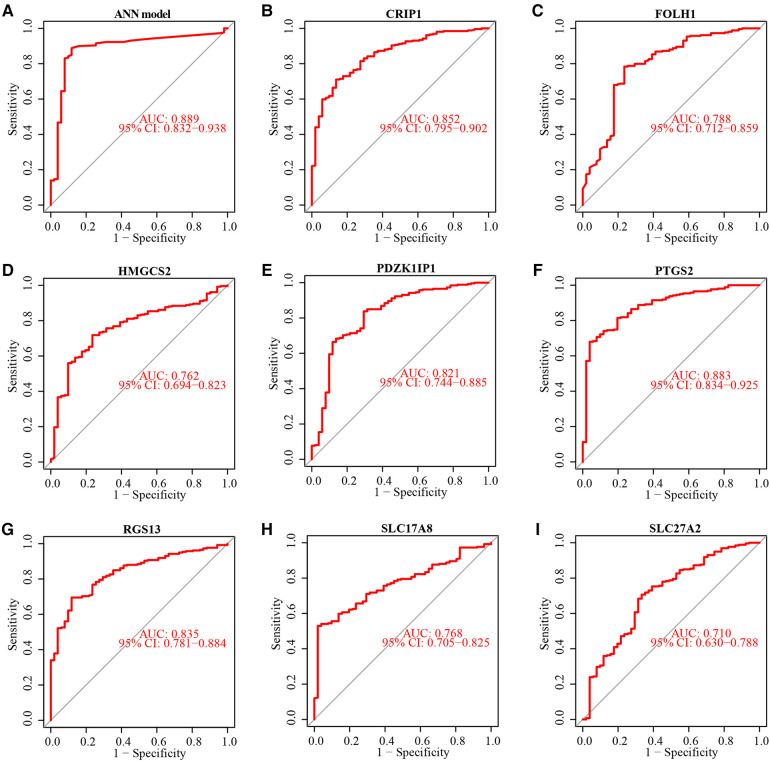
Performance evaluation of ANN Model and 8 diagnostic markers in testing subset. ROC curve showed diagnostic efficiency of ANN model in testing subset (**A**). ROC curve of diagnostic efficiency of 8 diagnostic markers in testing subset (**B–I**).

**Figure 7:**
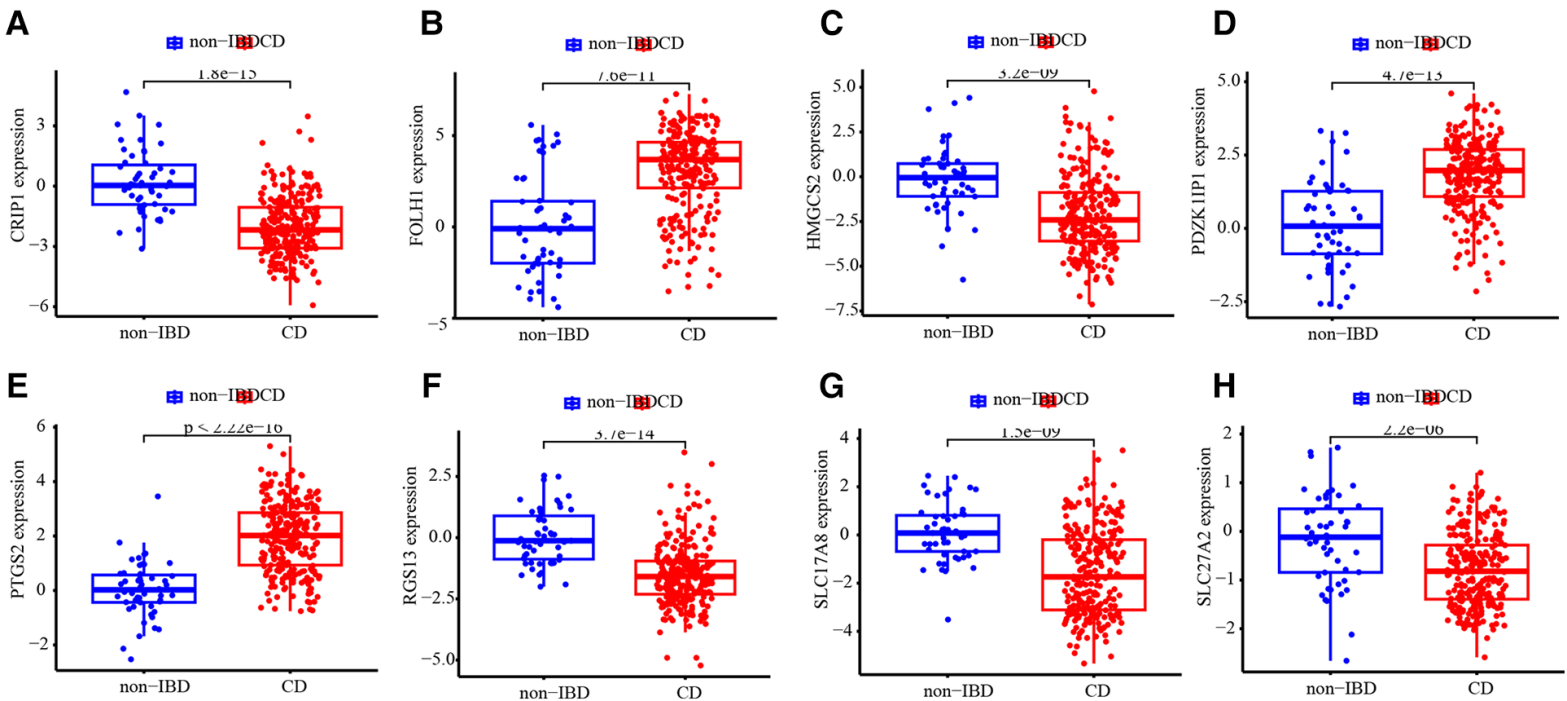
Expression level difference. Expression level difference of CRIP1 (**A**), FOLH1 (**B**), HMGCS2 (**C**), PDZK1IP1 (**D**), PTGS2 (**E**), RGS13 (**F**), SLC17A8 (**G**) and SLC27A2 (**H**) between CD and non-IBD controls.

The testing subset was used to assess the ability of the ANN model and 8 diagnostic markers for diagnostic. The accuracy of diagnosis prediction in testing subset is 86.7%. The performance of ANN model for diagnostic in testing subset was examined using ROC curves (Figure 6A). And ROC curves for each diagnostic marker were also produced. The AUC values (Figures 6B–I) and estimated expression level difference (Figure 7) of 8 diagnostic markers between CD and non-IBD controls in testing subset were calculated and compared. *P*-value <0.05 was considered significant.

### Immune infiltration analysis

**Figure 8:**
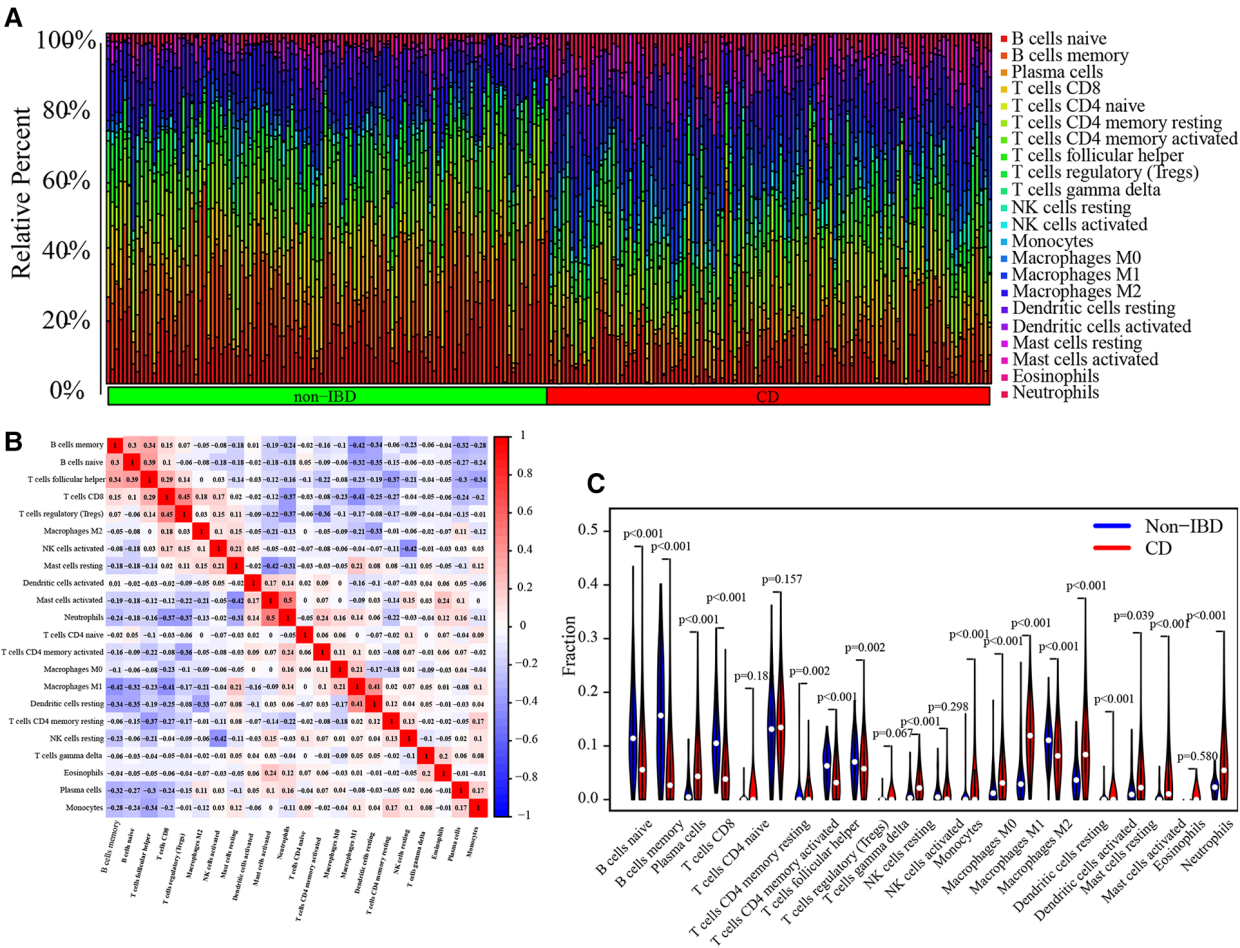
Analysis of immune-related cells. Bar chart of immune-related cells infiltration in each sample (**A**). Display of the correlation between immune-related cells (**B**). Violin diagram for difference analysis of 22 types of immune-related cells between pediatric CD and non-IBD (**C**).

**Figure 9:**
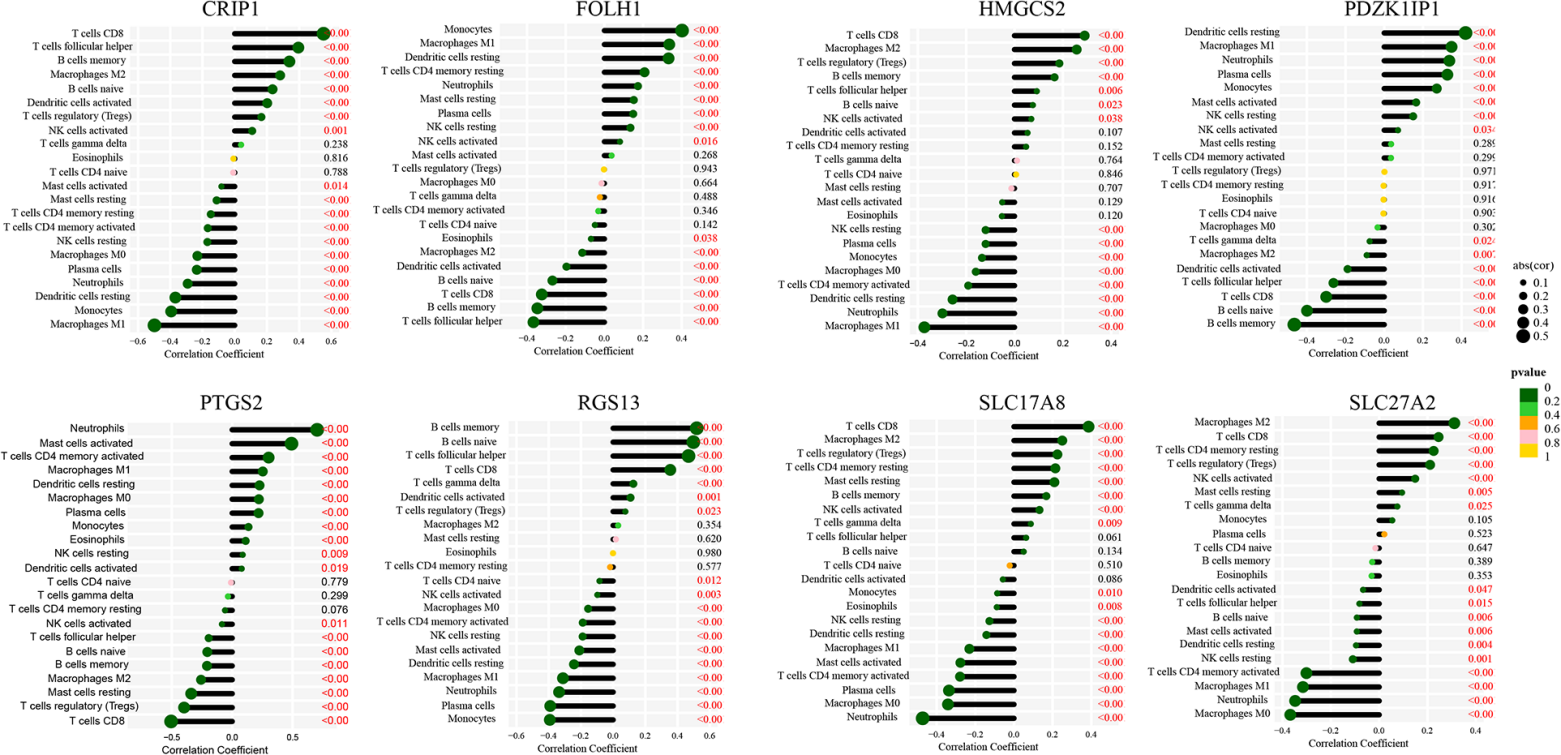
Association of immunity with diagnostic markers. Correlation between various immune cells and CRIP1 (**A**), FOLH1 (**B**), HMGCS2 (**C**), PDZK1IP1 (**D**), PTGS2 (**E**), RGS13 (**F**), SLC17A8 (**G**) and SLC27A2 (**H**).

The immune landscape between pediatric CD and non-IBD was explored using CIBERSORT algorithm based on the merged dataset (Figure 8A). The correlation analysis between immune cells showed that the negative correlation between memory B cells and M1 Macrophages (correlation coefficient = −0.42), activate Mast cells and resting Mast cells (correlation coefficient = −0.42), resting NK cells and activated NK cells (correlation coefficient = −0.42), M1 Macrophages and CD8 T cells (correlation coefficient = −0.41) was relatively strong. On the other hand, the positive correlation coefficient between CD8 T cells and regulatory T cells, which was 0.45, was the strongest (Figure 8B). According to the results, the proportion of activated Dendritic cells, resting Dendritic cells, M0 Macrophages, M1 Macrophages, activated Mast cells, resting Mast cells, Monocytes, Neutrophils, resting NK cells, Plasma cells and memory activated CD4 T cells were higher in pediatric CD, while the proportion of memory B cells, naive B cells, M2 Macrophages, CD8 T cells, follicular helper T cells and regulatory T cells were lower (Figure 8C). The correlation analyses between immune cells and diagnostic markers demonstrated that three types of immune cells (activated NK cells, CD8 T cells and gamma delta T cells) had a significant positive correlation with 5 markers. There was a significant positive correlation between activated NK cells and CRIP1, PDZK1IP1, FOLH1, SLC17A8 and HMGCS2. Gamma delta T cells had a significant positive correlation with CRIP1, RGS13, HMGCS2, SLC27A2 and SLC17A8. And CD8 T cells was positively correlated with CRIP1, RGS13, SLC27A2, SLC17A8 and HMGCS2. On the other hand, Eosinophils was negative correlated with 7 markers, including CRIP1, FOLH1, SLC17A8, PDZK1IP1, RGS13, SLC27A2 and HMGCS2 (Figure 9). The specific correlation between each diagnostic marker and immune cells is represented in detail (Supplementary Figure S1–S8).

## Discussion

Advances in machine learning and next-generation sequencing have enabled molecular diagnostic for complex diseases such as CD. Venkatapurapu SP et al. developed a hybrid mechanistic-statistical platform to predict outcomes and patient progress in Crohn's disease (38). Li L et al. analyzed biomarkers and constructed a classifier in prediction of Infliximab primary non-response for CD therapy (39).Though above models may assist therapy selection in clinical practice, they are limit for reliable and swift determination of diagnosis. Ostrowski J et al. identified the moderate discriminative power of transcriptional biomarkers for prediction of IBD clinical activity in pediatric populations (40).

The major aim of the present study consists in the setup of an ANN model for the diagnosis of pediatric CD based on gene expression profiling obtained from public GEO database. At first, a DEGs analysis between the CD and non-IBD groups was performed to identify 120 genes as pediatric CD related DEGs. The functional annotation indicated that DEGs were mainly enriched in some terms associated with immunity and inflammation, such as leukocyte mediated immunity, Complement and coagulation cascades, IL-17 signaling pathway, complement and coagulation cascades and cytokine receptor interaction. The results demonstrated that DEGs may involve in the inflammation and immune response of CD. Furthermore, 8 genes were identified as potential diagnostic biomarkers using machine learning algorithms including RF, LASSO, SVM-RFE and WGCNA. PDZK1IP1 is involved in the regulation of intestinal ion transport in IBD (41, 42). HMGCS2 contribute to increased ketogenesis and attenuates apoptosis and inflammation in intestinal pathology (43). PTGS2 is involved in the process of healing bowel wounds by regulating the production of prostaglandins (44). Above 3 biomarkers were all detected by 4 machine learning algorithms here. However, very little is known about the role of the other 5 identified genes in CD at present. It is reported that the expression levels of RGS13 in colon tissues associate with endoscopic remission after vedolizumab in IBD patients (45). FOLH1 can increase folic acid levels, which may promote proliferation of inflammatory cells (46). PTGS2 (AUC = 0.883) and CRIP1 (AUC = 0.852) showed moderate discriminative power in training and testing subsets, respectively.

Furthermore, an ANN diagnostic model was built based on 8 aforementioned biomarkers. The diagnostic performance of ANN diagnostic model and 8 diagnostic biomarkers were systematically evaluated. The model was able to provide an overall reliable accuracy when predicting diagnosis of pediatric CD in testing subset (86.8%). Compared with each diagnostic biomarker, the ANN diagnostic model had the best performance for diagnosis of pediatric CD. The ROC curve analysis performed subsequently could support this result. The ANN diagnostic model exhibited high sensitivity and specificity for diagnosis in both of training (AUC = 0.954) and testing subsets (AUC = 0.889).

Several studies demonstrated the essential role of intestinal immunity in both of gut defense and inflammatory mucosa damage (47–49). Immunosuppression and biologicals are the crucial therapies of CD. Immune cell infiltration analysis in our study showed that activated Dendritic cells, resting Dendritic cells, M0 Macrophages, M1 Macrophages, activated Mast cells, resting Mast cells, Monocytes, Neutrophils, resting NK cells, Plasma cells and memory activated CD4 T cells were enriched in CD. Previous research has demonstrated the augmented proportion of Neutrophils in mucosa of CD is positively correlated with disease severity (50). Memory activated CD4 T cells contribute to the pathogenesis of organ damage in autoimmune diseases, such as lupus nephritis, lupus encephalitis and neuropsychiatric lupus (51). Dendritic cells can initiate immune responses, control intestinal inflammation, and maintain tolerance. Defects in the regulation of Dendritic cells may lead to Crohn's disease (52).

Similarly, Monocytes also play essential roles in healthy and inflamed intestine (53). The macrophages can be distinguished by 3 subtypes, including inactivated M0 macrophages, classically activated M1 macrophages and alternatively activated M2 macrophages. They are all responsible for the early promotion and resolution of intestinal inflammation. M1 macrophages can stimulate inflammation, while M2 macrophages can antagonize inflammation and promote tissue repair (54). In present study, M0 Macrophages and M1 Macrophages are all enriched in CD. Hence, we speculated that intestinal macrophages may be associated with intestinal chronic inflammatory and finally structuring complications of CD. However, the proportion of memory B cells, naive B cells, M2 Macrophages, CD8 T cells, follicular helper T cells and regulatory T cells decrease in CD, which reflects the complexity of infiltration of immune cells. We found that resting Eosinophils had a significant negative correlation with 7 biomarkers. It may imply that resting Eosinophils and 7 biomarkers have antagonistic effects in the pathogenesis of CD. However, the potential meaning of the relationship between biomarkers and immune cells is not well elucidated. The expression of biomarkers may lead to intestinal inflammation by mediating immune cell infiltration in CD, which provides novel ideas and strategies for the study of treatment.

In the present study, LASSO, RF, SVM-RFE, WGCNA algorithms and ANN model were combined innovatively to develop a diagnostic model for pediatric CD. The model showed excellent diagnostic performance in a large text cohort. In addition, we explored the association of immunity with diagnostic markers and tried to demonstrate the rationality of diagnostic markers selection. The combination of biometric big-data and machine learning is ideal for accurate and early diagnosis in CD.

However, the present study has some drawbacks and limitations. First, there may be some bias in the research results because of the small sample size of pediatric CD in the GEO database. Secondly, due to the limitations of retrospective studies, prospective studies are needed to further elaborate the mechanism of some conclusions in our study. And finally, the conclusions of this study have not been verified by external data, which is needed to ensure the extrapolation and application of the conclusion.

## Conclusion

In conclusion, our study has constructed the diagnostic model of pediatric CD based on machine learning and explored the relation between pediatric CD and infiltration of immune cells. After identifying 8 diagnostic markers and constructing the diagnostic model of artificial neural network, we further explore the infiltration of immune cells in pediatric CD, and the association between diagnostic markers and immune-related cells. The results of our study can be expected to provide a basis for improving the early diagnosis and treatment of pediatric CD. However, for the clinical application of the results, further researches will be required in the future.

## Data Availability

The datasets presented in this study can be found in online repositories. The names of the repository/repositories and accession number(s) can be found in the article/**Supplementary Material**..
