# Supplementary figures and images for: Pediatric Crohn's disease diagnosis aid via genomic analysis and machine learning
Source: Front Pediatr. 2023 Mar 23;11:991247. doi: 10.3389/fped.2023.991247 (PMC10076664; doi:10.3389/fped.2023.991247)

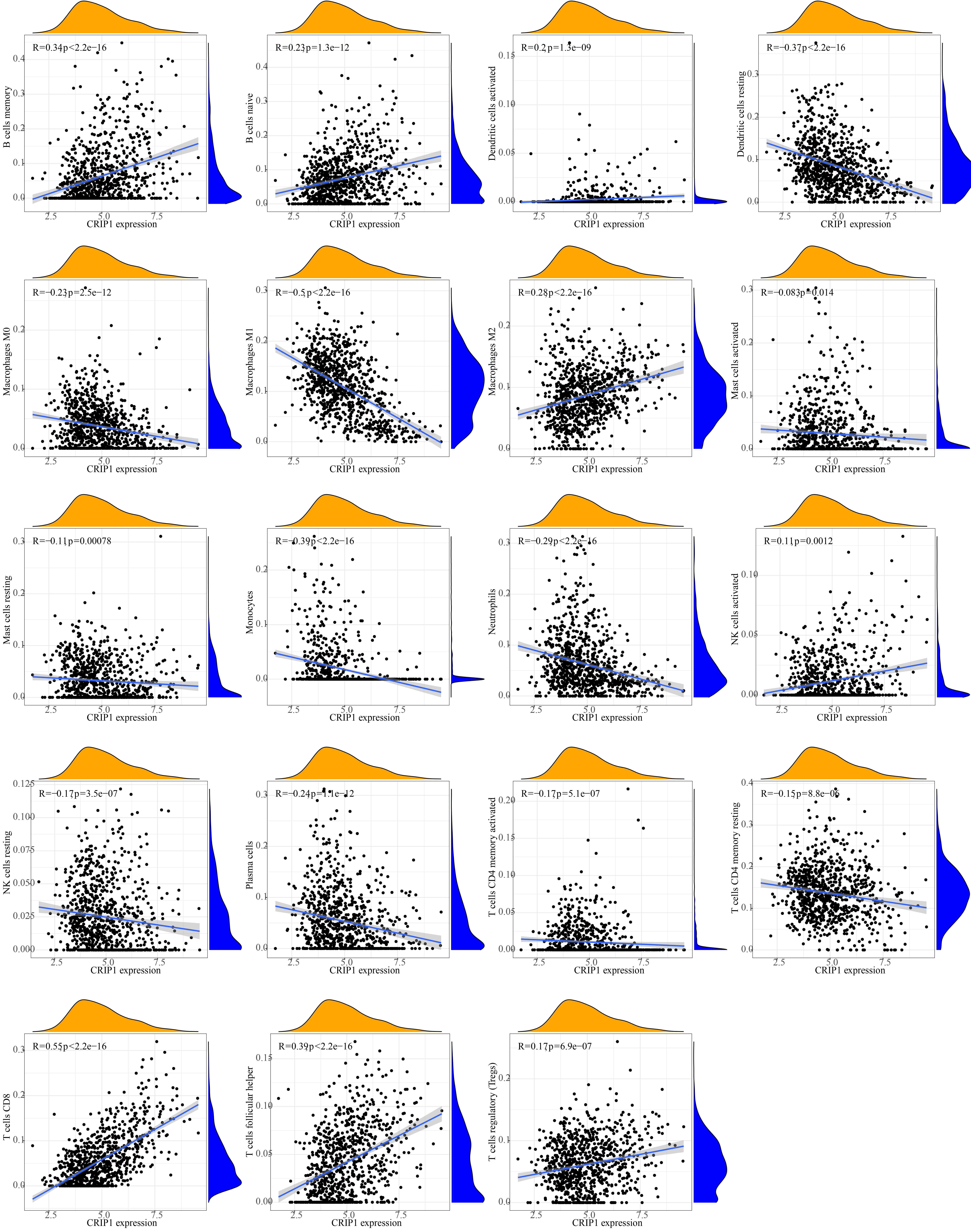

Supplement: Supplementary file 5 [file Image1.tif]

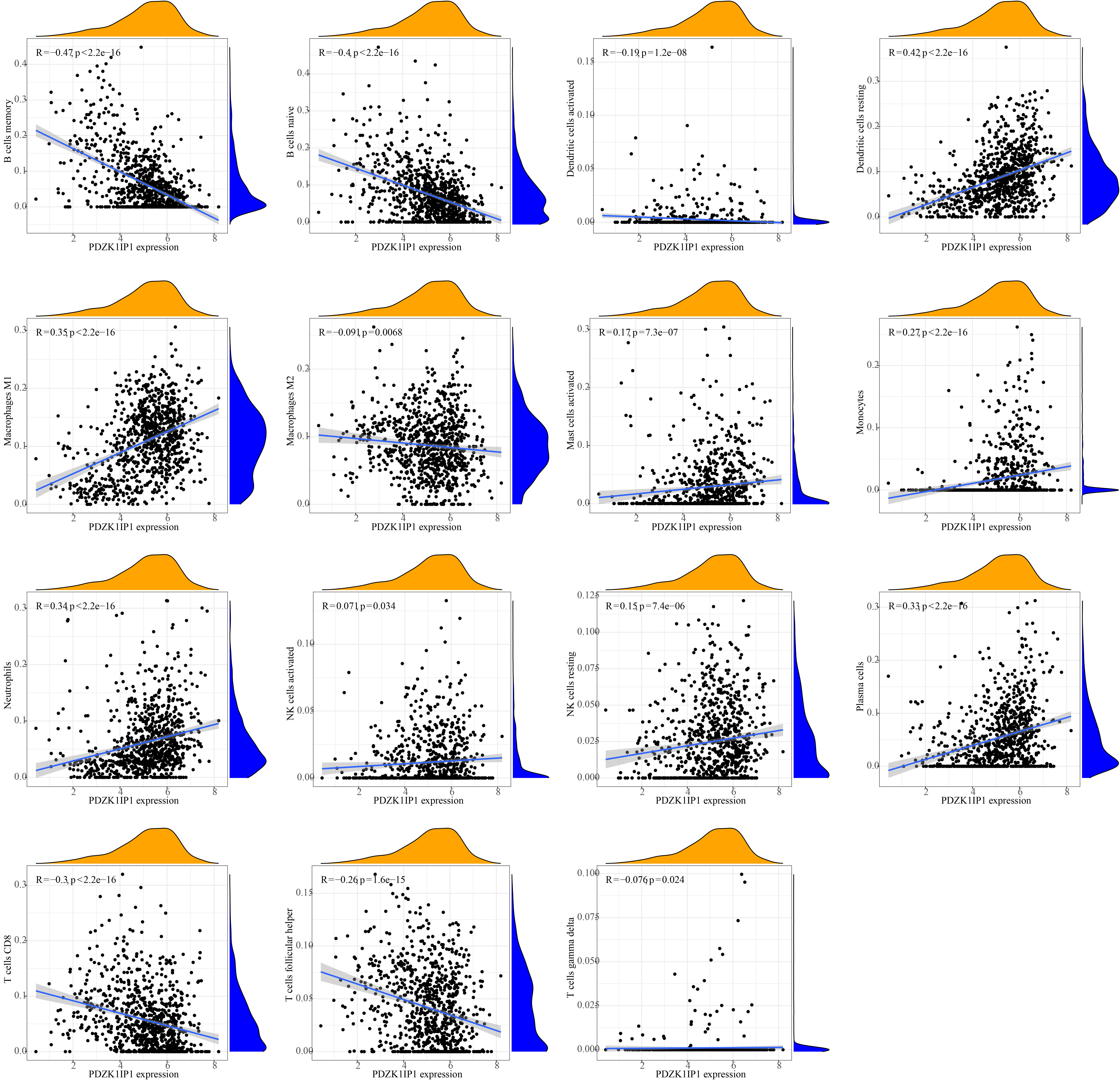

Supplement: Supplementary file 6 [file Image2.tif]

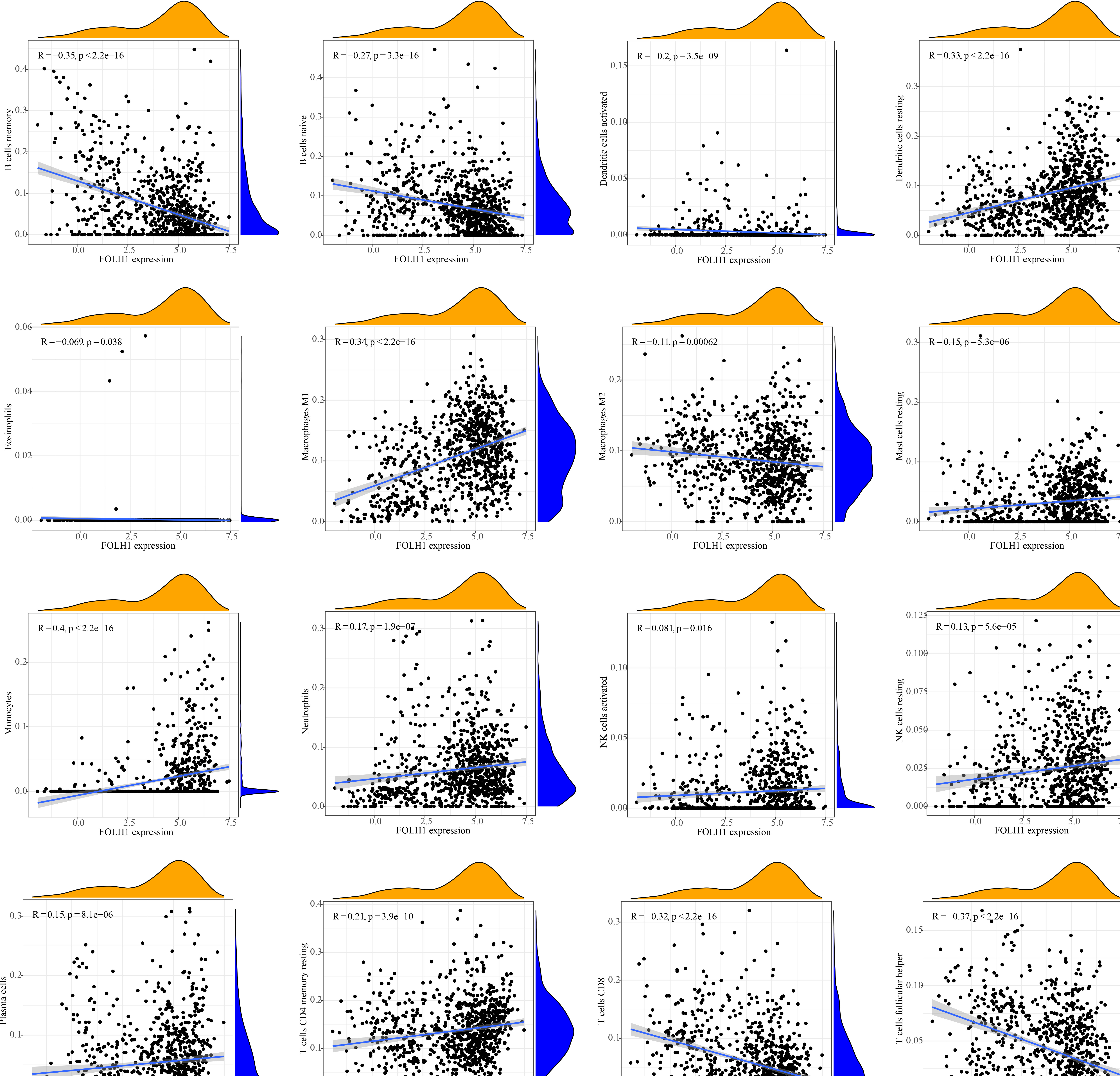

Supplement: Supplementary file 7 [file Image3.tif]

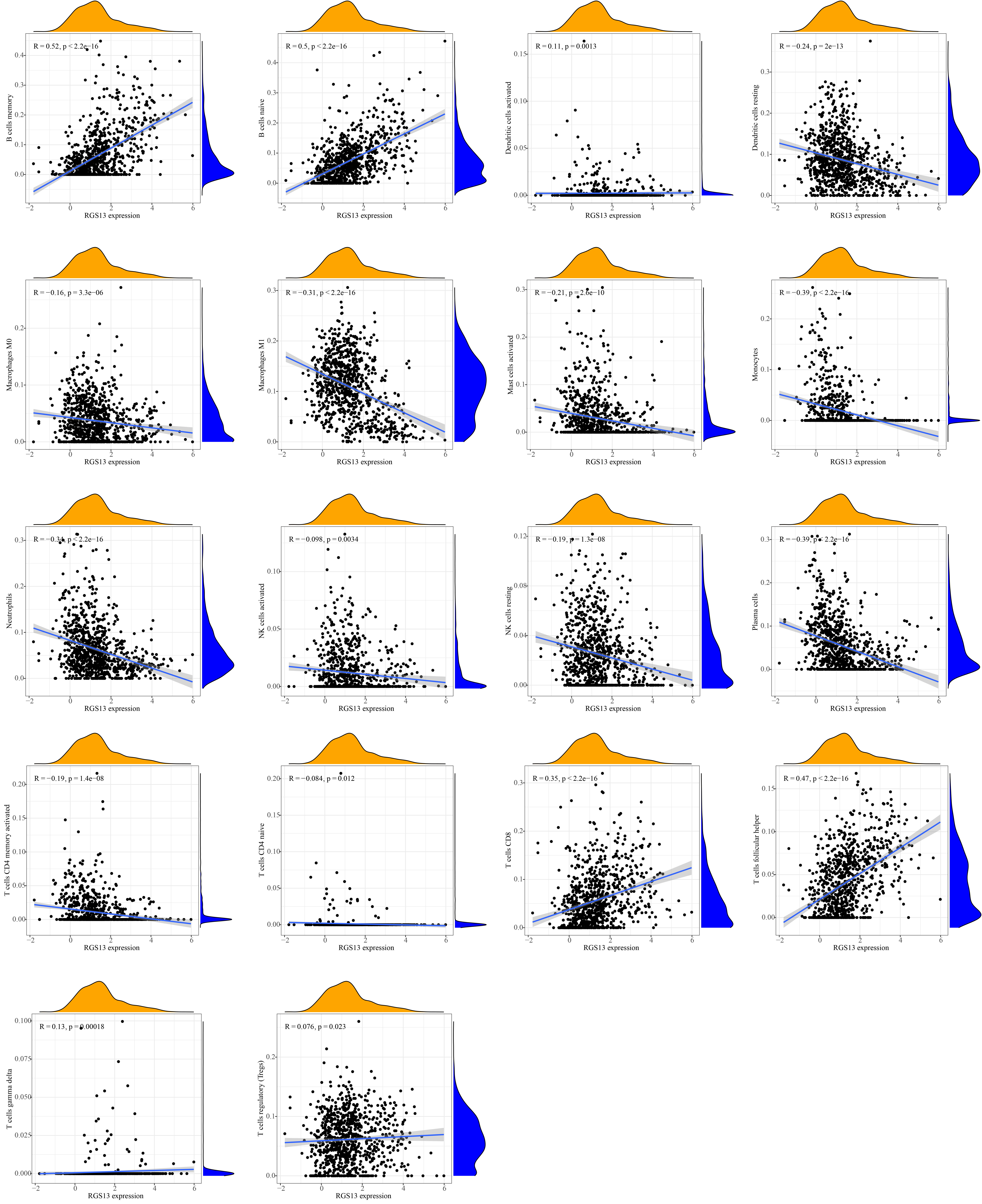

Supplement: Supplementary file 8 [file Image4.tif]

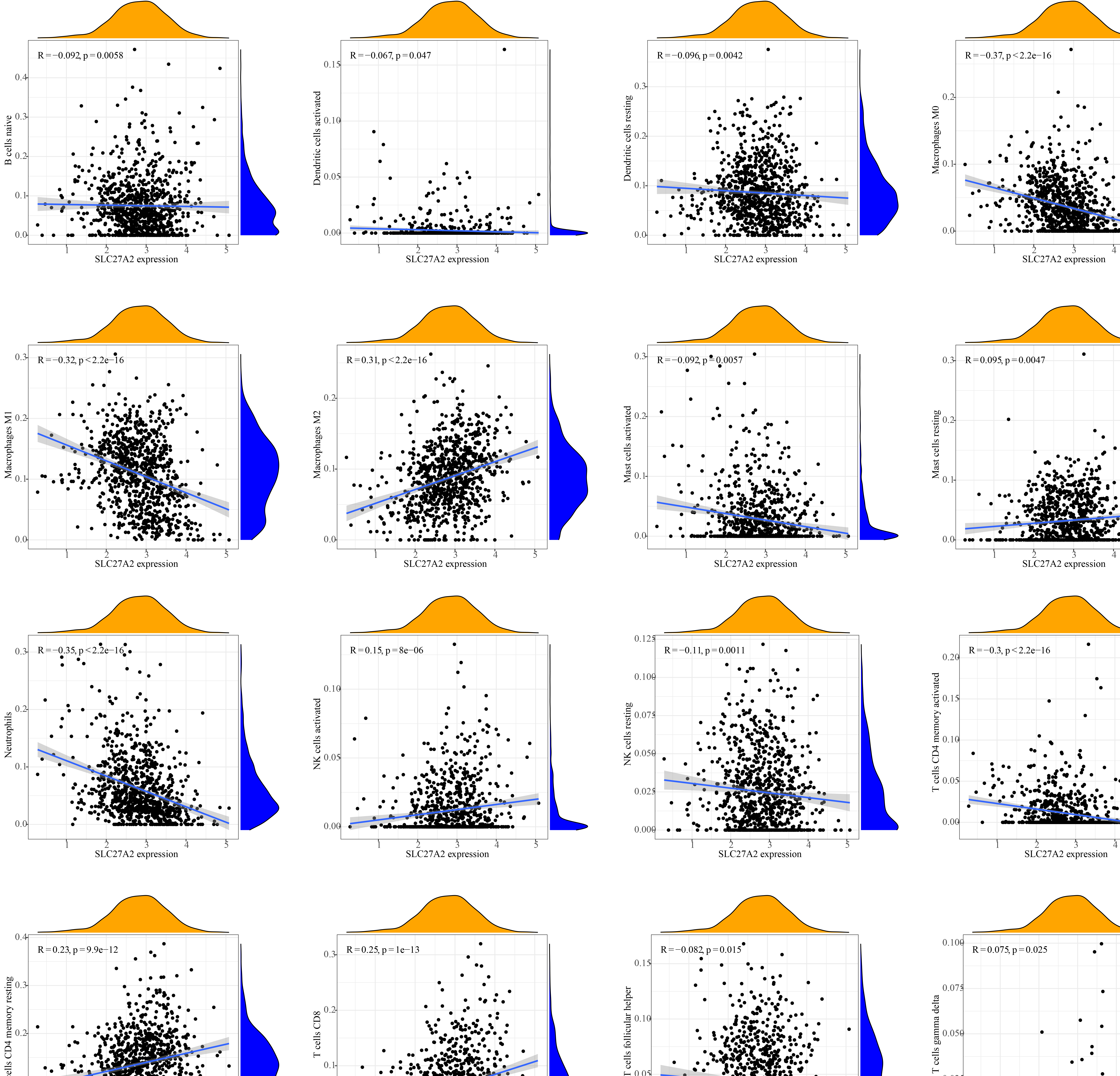

Supplement: Supplementary file 9 [file Image5.tif]

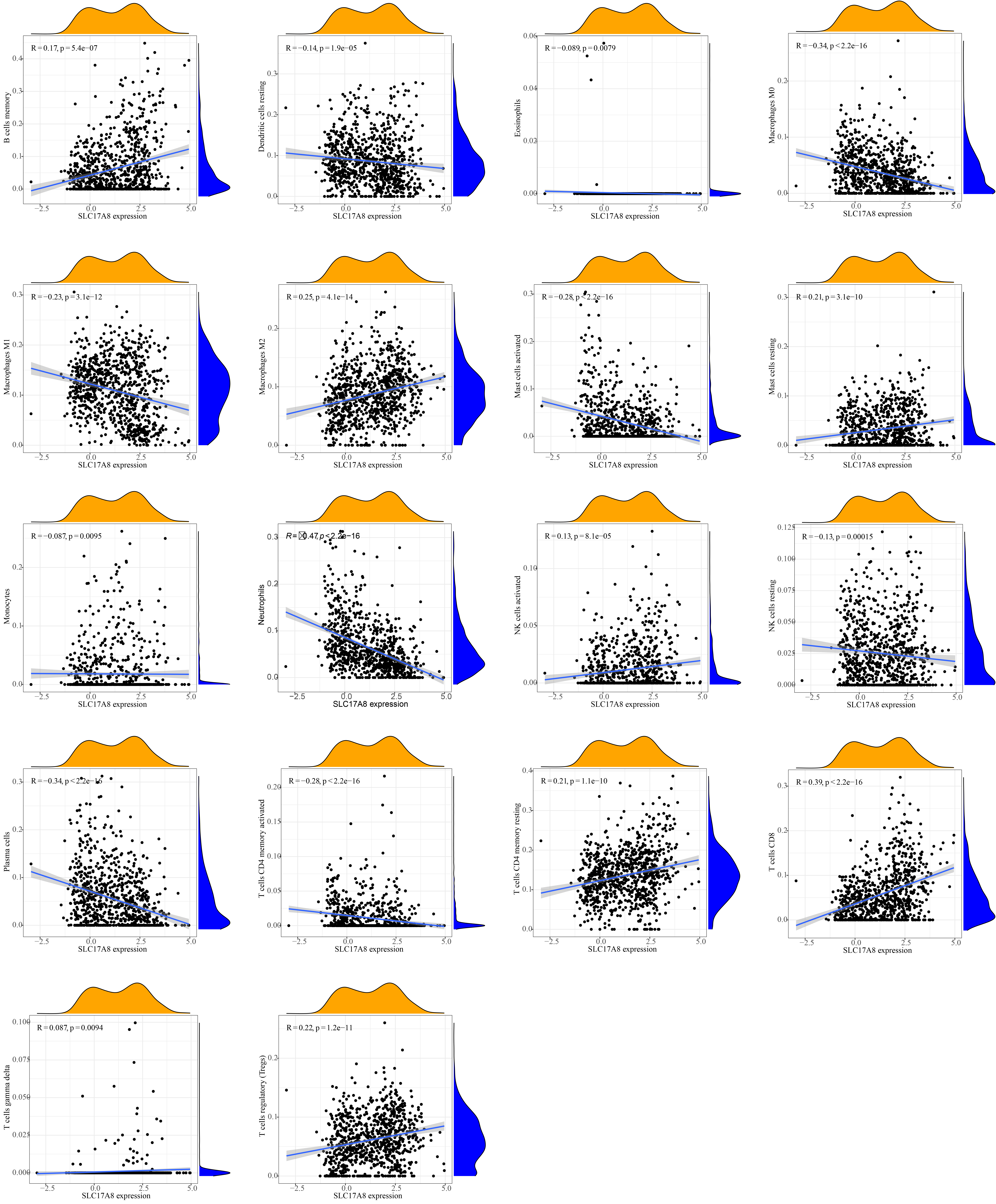

Supplement: Supplementary file 10 [file Image6.tif]

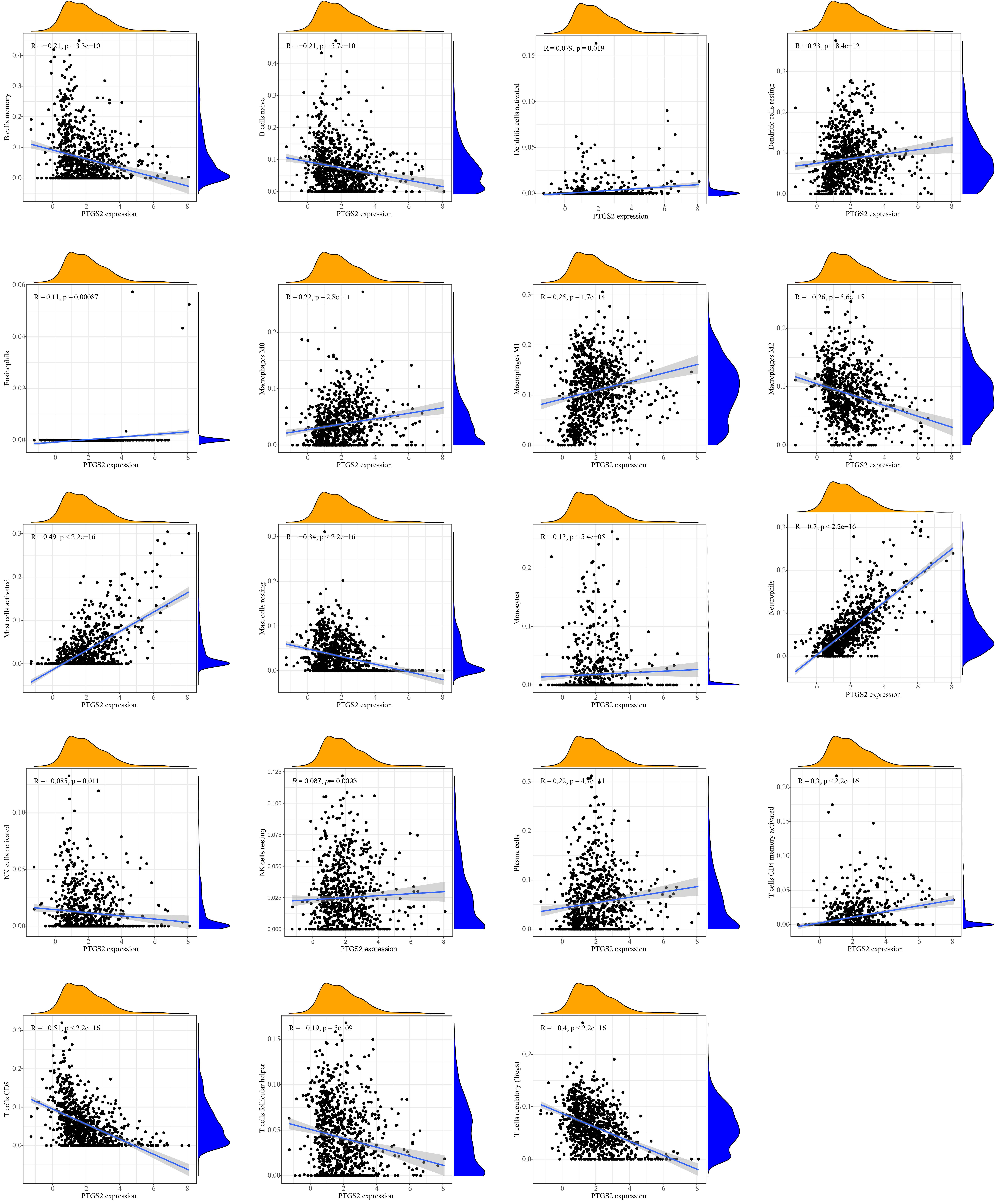

Supplement: Supplementary file 11 [file Image7.tif]

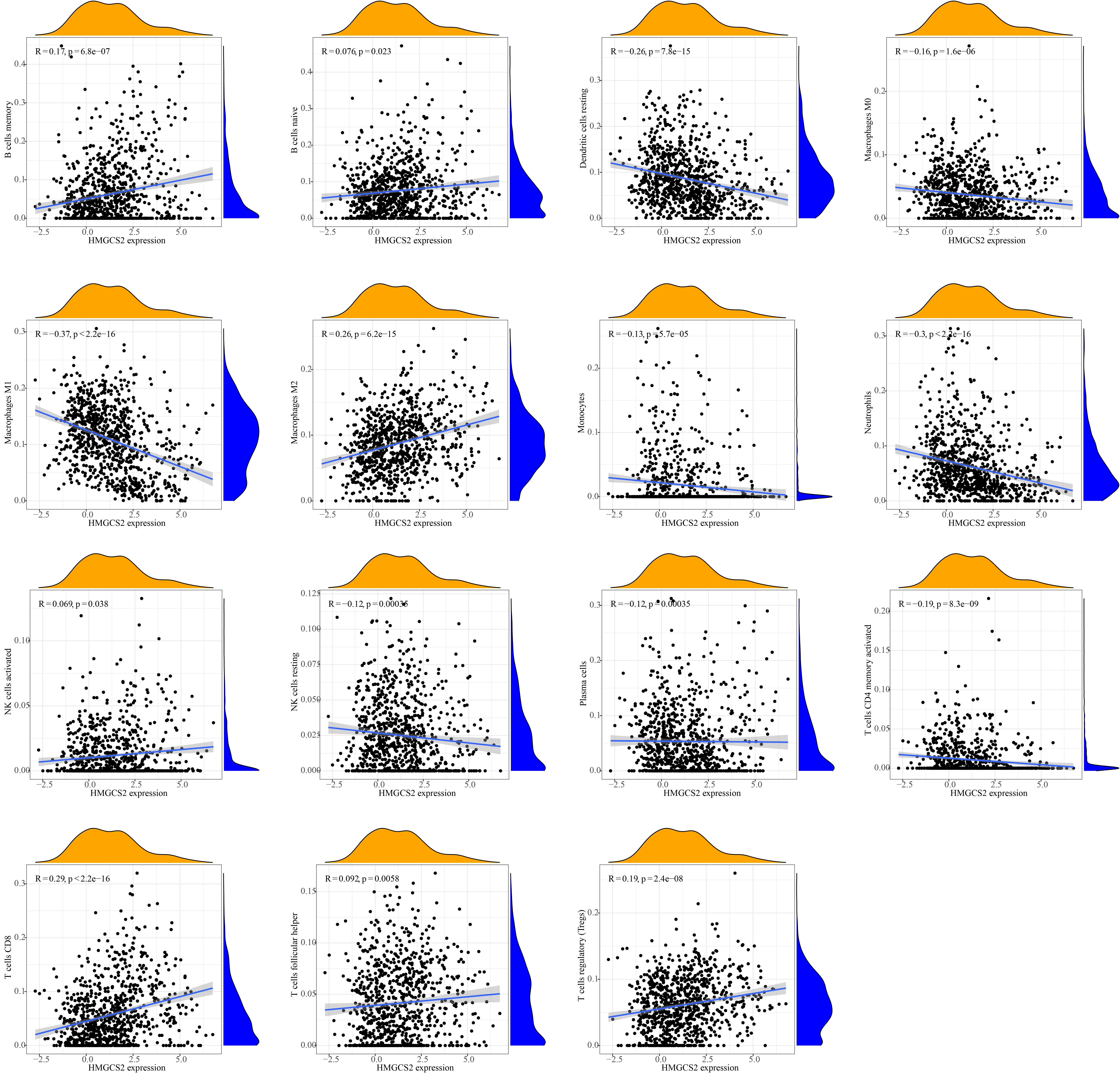

Supplement: Supplementary file 12 [file Image8.tif]
